# Activation of minority-variant *Plasmodium vivax* hypnozoites following artesunate + amodiaquine treatment in a 23-year old man with relapsing malaria in Antananarivo, Madagascar

**DOI:** 10.1186/1475-2875-12-177

**Published:** 2013-05-31

**Authors:** Voahangy Andrianaranjaka, Jessica T Lin, Christopher Golden, Jonathan J Juliano, Milijaona Randrianarivelojosia

**Affiliations:** 1 Institut Pasteur de Madagascar, BP 1274, Antananarivo 101, Madagascar; 2 Division of Infectious Diseases, University of North Carolina School of Medicine, Chapel Hill, NC, USA; 3 Harvard University Center for the Environment, Cambridge, MA, USA; 4 Madagascar Health and Environmental Research (MAHERY), Maroantsetra, Madagascar

## Abstract

In endemic areas, *Plasmodium vivax* relapses are difficult to distinguish from new infections. Genotyping of patients who experience relapse after returning to a malaria-free area can be used to explore the nature of hypnozoite activation and relapse. This paper describes a person who developed *P. vivax* malaria for the first time after travelling to Boriziny in the malaria endemic coastal area of Madagascar, then suffered two *P. vivax* relapses 11 weeks and 21 weeks later despite remaining in Antananarivo in the malaria-free central highlands area. He was treated with the combination artesunate + amodiaquine according to the national malaria policy in Madagascar. Genotyping by PCR-RFLP at *pvmsp-3α* as well as *pvmsp1* heteroduplex tracking assay (HTA) showed the same dominant genotype at each relapse. Multiple recurring minority variants were also detected at each relapse, highlighting the propensity for multiple hypnozoite clones to activate simultaneously to cause relapse.

## Background

In Madagascar, the combination of artesunate + amodiaquine (ASAQ) is recommended since December 2005 as the first-line treatment for uncomplicated and non-severe malaria, regardless of the parasite species involved. One of the important differences between *Plasmodium falciparum* and *Plasmodium vivax* is the formation of hypnozoites that can cause relapses after a course of treatment [1]. Such relapses have been described as clonal in origin in individuals returning to non endemic areas [2], but more recent evidence suggests that multiple low-frequency variants commonly arise during relapse [3,4]. In this report is described a case where sequential *P*. *vivax* malaria relapses in a patient living in Antananarivo, a malaria-free urban area, following ASAQ treatment comprised multiple minority-variant parasites, suggesting simultaneous reactivation of multiple hypnozoite clones.

## Case description

**Figure 1:**
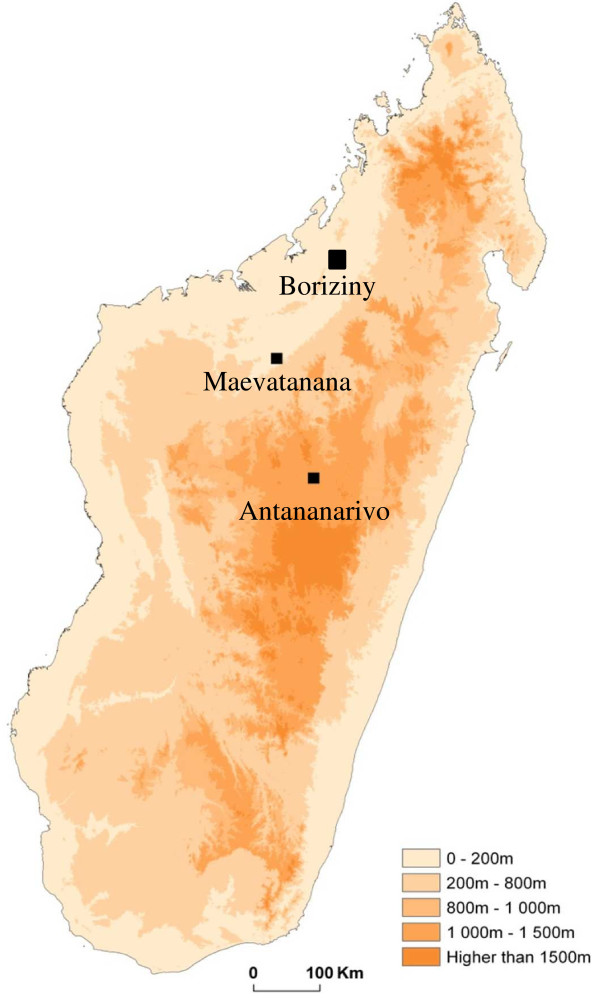
Map of Madagascar showing the geographical location of Boriziny and Antananarivo.

From May to November 2010, a 23 year old Malagasy man residing in Antananarivo travelled regularly to Boriziny (north-western Madagascar) as a ground transporter. He spent one to six nights there every one to two weeks without taking precautions to prevent mosquito bites. Antananarivo is the capital of Madagascar, situated in the central highlands at 1,200 m above sea level, and there is no malaria transmission throughout the city [5]. Boriziny (previously named Port Berger) is in a zone of tropical malaria transmission in north-western Madagascar, situated at less than 40 m above sea level (Figure 1). The subject returned from Boriziny on November 19, 2010 and reported having fever and chills on the evening of November 22, 2010. These symptoms were accompanied by sweating and fatigue. On the morning of November 23 (D0), he came to the malaria unit at the Institut Pasteur de Madagascar (IPM). Thick and thin peripheral blood smear were prepared, stained with Giemsa and microscopically examined as previously described [6]. Microscopy revealed *P*. *vivax* monoinfection with a parasitaemia of 6,687 parasites/μl. According to the malaria treatment policy in Madagascar, the patient was treated with a combination of artesunate + amodiaquine over three days (ASAQ Winthrop® for adults, artesunate 100 mg + amodiaquine 270 mg per tablet and 2 tablets per day). The first dose of ASAQ for D0 was administered under direct medical observation, with monitoring to insure that the patient did not vomit. He was given the ASAQ doses for D1 and D2 and was advised to return to the laboratory if malaria symptoms recurred. The subject spoke to staff from IPM on D1, D2 and D7 and reported that all symptoms were completely resolved. Prior to drug administration, as part of the national network for drug resistance surveillance established in Madagascar since 1999 (authorization no. 517-SAN/SG/DLMT/SLP and ethical clearance no. 013/04/SANPF/CAB), blood samples were collected on filter paper and kept at −20°C until use.

Following the first malaria episode on November 23, 2010, the patient remained in Antananarivo and did not travel back to Boriziny. However, on February 9, 2011 (D78), he developed fever and chills again and returned to the malaria unit at IPM. *Plasmodium vivax* monoinfection was confirmed by microscopy with a parasitaemia of 3,208 parasites/μl. Blood sample was collected on filter paper. The patient was again treated with ASAQ as described. The medical staff at IPM wanted to add primaquine to the treatment but none was available in Madagascar. After one week, the patient reported that symptoms had completely resolved.

Again, the patient remained in Antananarivo, but on April 22, 2011 (D150 with reference to the first attack in November 2010), he experienced similar symptoms as previously reported and returned to the the malaria unit at IPM. Microscopy was performed and *P*. *vivax* monoinfection was confirmed again with a parasitaemia of 63 parasites/μl. A blood sample was collected on filter paper, and the patient was treated with ASAQ for a third time. After a week, the patient reported that symptoms had completely resolved. Since November 2011, the subject has been living in France and has reported no further symptoms. Last contact was made with the subject on January 16, 2013.

### *Plasmodium vivax* genotyping

Parasite DNA extracted from filter paper blood samples was analysed to compare the genotypic profile of the *P. vivax* isolates collected from the patient during each of the three malaria episodes. Using an Instagen kit (Bio-Rad Laboratories Headquarters, France), DNA was extracted from the blood spots. Nested-PCR was used to confirm *P. vivax* monoinfection in the three samples from D0, D78 and D150 [7]. DNA from *P. vivax* collected from Maevatanana – a study site distant from Boriziny, was used as positive control. Human DNA from a blood donor from the HJRA University Hospital in Antananarivo was used as negative control. The lack of DNA contamination was checked using a blank (without DNA) in each run.

**Figure 2:**
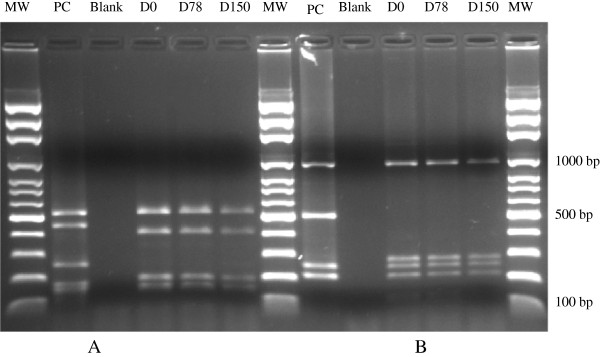
***Plasmodium vivax *merozoite surface protein-3α (*pvmsp-3α*) restriction fragment length polymorphism (RFLP) patterns after digestion with the restriction enzymes *Alu *I (2A) and *Hha *I (2B).** Lanes PC positive control, NTC negative control, Blank without DNA and D0, D78, D150 *P. vivax* samples from the patient. DNA size markers are shown in lanes labelled MW with sizes shown in basepairs (bp). MW: molecular weight (base pairs); PC: positive control; NTC: negative control; Blank: without DNA.

Next, *P. vivax* isolates were typed at *pvmsp-3α* as described elsewhere [8] to compare the D0, D78 and D150 genotypes. Using PCR-RFLP with AluI and HhaI restriction enzymes for *pvmsp-3α*, it was shown that the dominant strain of *P. vivax* causing the malaria attacks at D0, D78 and D150 was the same (Figure 2).

**Figure 3:**
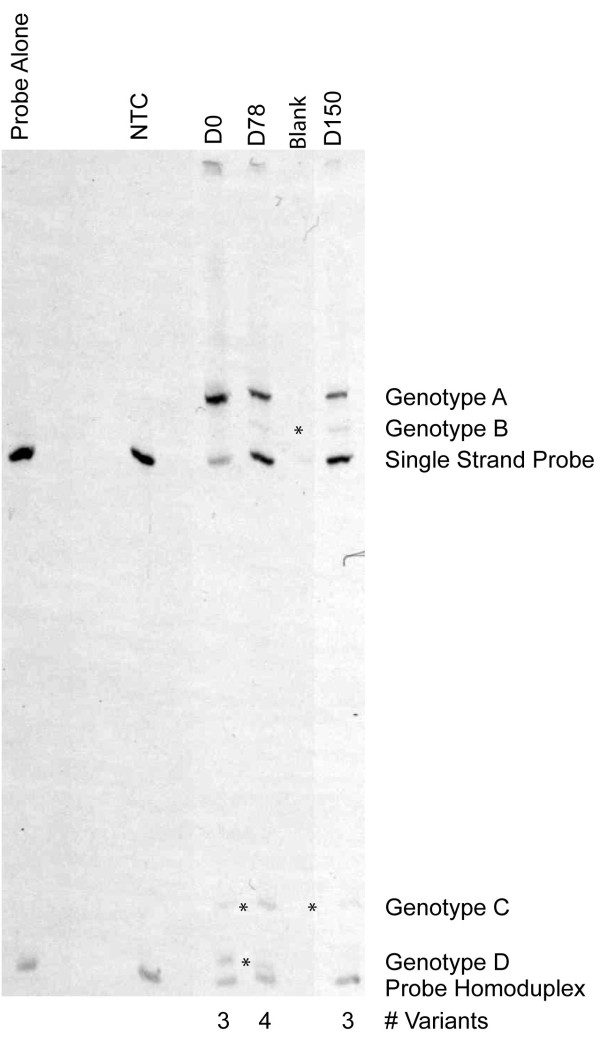
***Plasmodium vivax msp1 *heteroduplex tracking assay of the parasite isolates derived from the patient.** Each lane contains bands representing the sing-stranded HTA probe and probe homoduplex (seen in the NTC and probe alone lanes). The day of follow-up is indicated at the top of each lane. Unique pvmsp1 genotypes A-D were identified as bands that migrated differently from the single-stranded probe or probe homoduplex bands and are marked as asterisks. These represent heteroduplexes formed between the probe and amplified PCR product from each patient sample. Asterisks are placed between shared minority variants.

*Plasmodium vivax* infections are often polyclonal in nature, harbouring more than one genetic strain or variant at once [9,10]. The number of genetic variants found simultaneously within a host is referred to as the multiplicity of infection. To get a more nuanced understanding of the multiplicity of infection within the initial and relapsing isolates, a heteroduplex tracking assay (HTA) targeting the highly variable *P. vivax* merozoite surface protein 1 gene (*pvmsp-1)* as previously described was used [3]. It was demonstrated that the initial as well as two subsequent malaria episodes each harboured multiple *pvsmp1* variants by HTA, labelled Genotype A-D in Figure 3, yielding multiplicity of infections (MOIs) of 3–4. With each episode, genotype A remained the dominant genotype, as implied by the darkness of the band, while 2–3 other genotypes were detected as minority variants. On D0, genotype C represents a minority variant that recurred both 11 and 21 weeks later on D78 and D150. Genotype D, also found at D0, recurred at D78, but was not detected at D150. Finally, genotype B was not appreciated in the initial isolate, but appeared at D78 and recurred at D150 (Figure 3). Taken together, this pattern of multiple relapsing variants suggests that polyclonal *P. vivax* infections reactivate hypnozoites in a multiclonal fashion. While genotype B appeared as a novel variant at D78, possibly suggesting a new infection from a mosquito inoculation, the clinical history of the patient combined with the recurrence of genotype B at D150 strongly suggest that genotype B was present, but not appreciated in the initial D0 infection and achieved greater patency in the relapsing infections. Because it is not certain that this is the subject’s first bout of malaria, another possibility is that genotype B represents a reactivated latent hypnozoite from a prior *P.* vivax infection, preceding November 2010.

## Concluding remarks

The case reported herein involves *P. vivax* malaria imported from the coastal area to the central highland area in Madagascar. Repetitive ASAQ treatment administered according to the national malaria treatment policy was effective in treating the acute illness in the patient but relapses occurred twice. Parasitaemia was lower at each subsequent attack. *Pvmsp-3α* genotyping indicated that the same major strain was present during the three consecutive attacks. However, such PCR-based genotyping can miss other genotypes also present in low numbers. The HTA analysis suggests that multiple other clones were involved in the relapse.

*Plasmodium vivax* malaria is characterized by relapses after resolution of the primary infection, derived from activation of dormant hypnozoites in the liver [11]. The efficacy of ASAQ has been proven for treating uncomplicated *P. falciparum* malaria [6] and *P. vivax* malaria (Randrianarivelojosia, personal communication) in Madagascar. Regarding the case reported in this paper, *P. vivax* malaria occurring more than 70 days post-treatment in a patient living in the central highland does not indicate recrudescence due to ASAQ treatment failure; rather, it is indicative of typical relapse. *Plasmodium vivax* is the second most prevalent species of human malaria parasite behind *P. falciparum* in Madagascar, but it occurs in less than 5 %; of biologically confirmed malaria cases [12,13]. Thus, it is logical to give ACT universally for all *Plasmodium* species whether *P. falciparum* or *P. vivax*. However, *P. vivax*-specific treatment of hypnozoites in the form of primaquine for radical cure is not the policy in Madagascar. A subset of patients will suffer relapse as in this gentleman. From a public health perspective, it would be important eventually to take into account the use of primaquine to eradicate dormant *P. vivax* hypnozites and prevent these relapses.

To some degree, the level of genetic complexity seen in malaria infections is associated with transmission intensity. Even though *P. falciparum* dominates malaria transmission in Madagascar, the detection of multiple minority-variants of *P. vivax* in the reported case suggests that genetic diversity of *P. vivax* parasites exists to the extent that polyclonal *P. vivax* infections are not rare. None of the minority variants detected evolved into the predominant strain at relapse. However, the detection of these minority variants allowed a more nuanced appreciation of the mechanisms of hypnozoite activation within a polyclonal infection. Simultaneous hypnozoite activation and relapse of multiple clones promotes genetic diversity in the parasite population as whole, and such relapse may be an important mechanism for maintaining high *P. vivax* genetic diversity even in areas of relatively low transmission. Furthermore, the ability to detect multiple recurring strains of the same type can lend further evidence that a recurrent malaria episode indeed represents relapse rather than new infection.

## Consent

In line with any activities part of the national network for malaria drug resistance surveillance (ethical clearance no. 013/04-SANPF/CAB on January, 21, 2004), written informed consent was obtained from the patient for blood and information collection, for follow up, and for publication of this report.

## Competing interests

The authors declare that they have no competing interests.

## Authors’ contributions

All authors contributed equally to preparing the final version of the manuscript. VA, JL, JJ and MR were in charge of the parasite genotyping and the microscopy. MR and JJ are guarantors of the paper.
